# Comparison of the miRNA profiles in HPV-positive and HPV-negative tonsillar tumors and a model system of human keratinocyte clones

**DOI:** 10.1186/s12885-016-2430-y

**Published:** 2016-07-04

**Authors:** Zuzana Vojtechova, Ivan Sabol, Martina Salakova, Jana Smahelova, Jiri Zavadil, Lubomir Turek, Marek Grega, Jan Klozar, Bohumir Prochazka, Ruth Tachezy

**Affiliations:** Department of Genetics and Microbiology, Faculty of Science, Charles University in Prague, Prague, Czech Republic; Department of Immunology, Institute of Hematology and Blood Transfusion, U Nemocnice 2094/1, Prague 2, CZ-12820 Czech Republic; Molecular Mechanisms and Biomarkers Group, International Agency for Research on Cancer, Lyon, France; Veterans Affairs Healthcare System and Department of Pathology, University of Iowa, Iowa City, IA USA; Department of Pathology and Molecular Medicine, 2nd Faculty of Medicine, Charles University in Prague, Prague, Czech Republic; Department of Otorhinolaryngology and Head and Neck Surgery, 1st Faculty of Medicine, Charles University in Prague, Motol University Hospital, Prague, Czech Republic

**Keywords:** Human papillomavirus, miRNA, Tonsillar tumor, Head and neck cancer

## Abstract

**Background:**

Better insights into the molecular changes involved in virus-associated and -independent head and neck cancer may advance our knowledge of HNC carcinogenesis and identify critical disease biomarkers. Here we aimed to characterize the expression profiles in a matched set of well-characterized HPV-dependent and HPV-independent tonsillar tumors and equivalent immortalized keratinocyte clones to define potential and clinically relevant biomarkers of HNC of different etiology.

**Methods:**

Fresh frozen tonsillar cancer tissues were analyzed together with non-malignant tonsillar tissues and compared with cervical tumors and normal cervical tissues. Furthermore, relative miRNAs abundance levels of primary and immortalized human keratinocyte clones were evaluated. The global quantitation of miRNA gene abundance was performed using a TaqMan Low Density Array system. The confirmation of differentially expressed miRNAs was performed on a set of formalin-fixed paraffin-embedded tumor samples enriched for the tumor cell fraction by macrodissection.

**Results:**

We defined 46 upregulated and 31 downregulated miRNAs characteristic for the HPV-positive tonsillar tumors and 42 upregulated miRNAs and 42 downregulated miRNAs characteristic for HPV-independent tumors. In comparison with the expression profiles in cervical tumors, we defined miR-141-3p, miR-15b-5p, miR-200a-3p, miR-302c-3p, and miR-9-5p as specific for HPV induced malignancies. MiR-335-5p, miR-579-3p, and miR-126-5p were shared by the expression profiles of HPV-positive tonsillar tumors and of the HPV immortalized keratinocyte clones, whereas miR-328-3p, miR-34c-3p, and miR-885-5p were shared by the miRNA profiles of HPV-negative tonsillar tumors and the HPV-negative keratinocytes.

**Conclusions:**

We identified the miRNAs characteristic for HPV-induced tumors and tonsillar tumors of different etiology, and the results were compared with those of the model system. Our report presents the basis for further investigations leading to the identification of clinically relevant diagnostic and/or therapeutic biomarkers for tumors of viral and non-viral etiology.

**Electronic supplementary material:**

The online version of this article (doi:10.1186/s12885-016-2430-y) contains supplementary material, which is available to authorized users.

## Background

Head and neck cancer (HNC) mostly involves a group of squamous cell carcinomas (HNSCC) which arise from the epithelial cells of the mucosal lining in the head and neck region. HNSCC belong among the six most common cancers worldwide [1]. The most important risk factors are smoking and alcohol consumption; however, HPV infection is also recognized as another primary cause of HNC. The proportion of HPV-caused HNC varies around the world; in the USA about 40–80 % of oropharyngeal cancers are associated with the presence and expression of high-risk HPV (HR-HPV), whereas in Europe, this percentage ranges from 90 % in Sweden to less than 20 % in countries with high consumption of tobacco [2]. In the Czech Republic, 68 % of the oropharyngeal tumors are HR-HPV positive [3, 4]. Recently, an increasing incidence of oropharyngeal cancers associated with HPV has been reported in several countries [5–7]. The most prevalent mucosal HR-HPV in HNC is HPV16 occurring in up to 90 % of HPV-positive HNC [8, 9].

Although patients with HR-HPV associated HNC often present with more advanced disease, they have a remarkably better prognosis and overall survival [9, 10]. The prognostic advantage of HNC tumors etiologically linked to HPV could potentially lead to modified treatment regimens. Besides, oropharyngeal tumors are usually not detected in the early stages of the disease, most likely due to the location of the lesions in the tonsillar crypts, and the marker of early cancer formation might allow earlier treatment and improved prognosis. Meanwhile, patients start to be treated in the late stages of disease and their prognosis is relatively poor. Unlike cervical cancer which is nearly always associated with HR-HPV infection, virally and non-virally induced HNC represent a unique model to study cancer in the same localization that is caused by distinct molecular mechanisms.

MicroRNAs (miRNAs) are a class of short single-stranded non-coding RNAs. MiRNAs play an important role in post-transcriptional regulation of gene expression by inducing target mRNA degradation or by repressing the translation via binding to the 3´-untranslational region of their target mRNAs [11]. The regulation of miRNA expression has been demonstrated to play a key role in the development, cell growth, and differentiation processes in a variety of eukaryotic organisms. The expression of miRNAs is deregulated in human cancer and their abundance profiles are often specific for tumors of different origin where they can serve as oncogenes or tumor suppressors. It has been shown that both normal and cancer tissues have specific miRNA expression signatures and show differential expression across tumor types [12]. In previous studies, it has also been demonstrated that miRNAs are promising prognostic and diagnostic biomarkers of human cancers [13–16].

Several studies focused on miRNA profiling in HNC have been published in recent years. Distinct miRNA profiles were shown in head and neck cancer cell lines [17] as well as in tumor tissues compared with normal tissues [18–20]. However, to date only a few studies analyzing the miRNA profile in HNC with regard to HPV presence have been published. HPV-positive and HPV-negative HNSCC cell lines were studied by Wald et al. [21]. Lajer et al. profiled HPV-positive and HPV-negative oral, pharyngeal, and oropharyngeal (OSCC, PSCC, OPSCC) and cervical squamous cell carcinomas [22, 23] and identified the “HPV core” miRNAs.

The aim of our study was to characterize the expression profiles of miRNAs in well-characterized tonsillar tumors and define miRNAs characteristic for HPV-dependent and HPV-independent tonsillar tumors. Cervical tumors were also evaluated as a positive control of HPV-associated tumors, allowing us to identify miRNAs specific for HPV-induced tumors. Furthermore, we compared the results of miRNA expression profiles with the model system of isogenic primary human keratinocyte clones immortalized by HPV or human telomerase gene. We identified a group of miRNAs specific for HPV-induced tumors and a group of miRNAs specific for tonsillar carcinomas. The expression of the most differentially expressed miRNAs was confirmed on a large set of macrodissected tonsillar tumors by quantitative real-time PCR (RT qPCR). Finally, we have identified several miRNAs as potential prognostic markers but their significance has to be determined in a larger sample set. Our results serve as a starting point for the identification of useful and clinically relevant biomarkers of HNC tumors of HPV-related and HPV-independent etiologies.

## Methods

### Clinical samples

All tonsillar tumor samples were obtained in the scope of the ongoing study from patients treated at the Department of Otolaryngology and Head and Neck Surgery, 1st Faculty of Medicine, Charles University and Motol University Hospital, Prague in the period from 2005 to 2007. The study set, i.e. 23 fresh frozen (FF) tumor samples and 64 formalin-fixed paraffin-embedded (FFPE) tumor samples, was selected based on the virological and immunohistochemical characteristics of the FFPE tissue samples in our ongoing studies (p16, p53, HPV DNA, and HPV E6 mRNA) [24] and the availability of fresh frozen material. Normal tonsillar tissue samples (*N* = 5) were collected from patients who underwent tonsillectomy for non-malignant conditions. These patients were gender and age matched to the study patients with tonsillar tumors. Cervical samples, i.e. normal cervical tissues (*N* = 2) and cervical tumors (*N* = 5), were collected from patients treated at the Department of Obstetrics and Gynecology, 2nd Faculty of Medicine, Charles University and Motol University Hospital, Prague in 2012. All patients enrolled in the study signed the informed consent form. The study received official institutional and ethical approval from the Motol University Hospital and Institute of Hematology and Blood Transfusion.

For patients from whom FF tonsillar tumor samples were obtained, data on demographics and clinical pathological characteristics were completed for each patient and collected by a questionnaire. All data are summarized in Additional file 1: Table S1. All patients were prospectively followed up.

The sampling and tissue handling for tonsillar tumor samples in the ongoing study has been described before [25]. Sections of fresh frozen tumor tissues were cut on a cryostat, and the number of tumor cells was determined by a pathologist. The samples of non-malignant tonsils were taken during the surgery and were snap frozen in liquid nitrogen immediately after removal and stored at −80 °C. Samples of normal cervical tissue and cervical tumors were taken during the surgery, stored and transported in RNAlater (Life Technologies, USA) transport medium, and processed within 1 week. FFPE samples were macrodissected. The area with the tumor was labelled by an expert pathologist on a hematoxylin stained slide and transported to the laboratory. The tissue from the labelled area was scraped off and used for immediate RNA extraction.

A set of nine isogenic primary human foreskin keratinocyte clones and the w12 cell line (derived from cervical carcinoma containing extrachromosomal HPV16) were kindly provided by the collaborating laboratories at the University of Iowa, USA. The preparation of the clones has been previously described [26, 27]. Five of the foreskin keratinocyte clones were immortalized by HPV16, two were immortalized by the telomerase gene, and the rest were primary keratinocyte cells. The cells were stored at -80 °C and used directly for the isolation of nucleic acids. The characteristics of analyzed clones and cell lines are summarized in Table 1.

**Table 1:** Specification of keratinocyte clones and cell lines

Type of cells	Specification	Number
HPV16 immortalized human keratinocyte clones	integrated	3
	extrachromosomal	1
	mixed	1
HPV16-positive original w12 cell line	extrachromosomal	1
primary human keratinocytes	HPV-negative	2
hTERT immortalized human keratinocyte clones	HPV-negative	2

### Processing of samples

DNA from all FF tonsillar tissues and cervical tissues was isolated using the QIAamp DNA Mini kit (Qiagen, Germany) and total RNA was extracted by the miRVana kit (Life Technologies, USA) according to the manufacturer's protocol. For better yield, only RNA was isolated from the cell lines. Both DNA and RNA of FFPE samples were simultaneously extracted from four 10-μm sections enriched for the tumor cells by macrodissection using the Ambion RecoverAll™ total nucleic acid isolation kit for FFPE according to the manufacturer's protocol (Applied Biosystems, USA).

The detection and typing of HPV DNA was performed by a modified PCR method with broad spectrum primers BSGP5+/6 + bio specific for the L1 region and reverse line blot hybridization as previously described [28]. RNA concentration and quality were measured by Experion chip electrophoresis (Bio-Rad, USA). For the analysis of microarrays, only FF samples with a RIN (RNA integrity number) higher than seven were used. The expression of HPV16 E6 mRNA was assessed by RT PCR which amplifies the most abundant splice variant E6*I with a length of 86 bp as described before [29]. The status of the HPV genome in FF clinical samples as well as in the cell lines was evaluated by the mapping of E2 integration breakpoint and APOT assay as described recently [25].

### TaqMan Low Density Array (TLDA) analysis

The global quantitation of miRNA gene expression was performed using the TaqMan® Array Human MicroRNA A + B Cards Set v3.0 (Life Technologies, USA) containing a total of 384 TaqMan® MicroRNA Assays and controls per card. Each array contains three assays of endogenous controls (RNU48, RNU44, and RNU6B) and one assay as a negative control.

Overall, we analyzed 35 clinical samples and 12 cell lines. First, 1000 ng of total RNA of each sample were reverse transcribed using the TaqMan® MicroRNA Reverse Transcription Kit and Megaplex™ RT Primers specific for each card (both Life Technologies, USA) according to the manufacturer's instructions. The TLDA cards were analyzed on the Applied Biosystems 7900HT Real-Time PCR System.

### Data analysis

For data processing and evaluation, we used the SDS 2.4 and the ExpressionSuite v1.0.1. software (Life Technologies, USA). Ct values for miRNAs identified by automated thresholding were exported separately for cards A and B. From the detected Ct values, the RQ (relative quantity) was calculated using the 2^-Ct formula. The data were further processed by the GeneSpring GX11 software (Agilent). Based on the per-sample measurement counts (hundreds), we selected the 50th percentile shift to perform within-sample normalization and applied it globally across the data sets. For comparison of two or three groups in the censored analyses, T-test or ANOVA, in combination with the Pavlidis Template Matching tool (TIGR TM4 suite) was used. The *P*-value (*P* < 0.05) and fold-change (FC> 1.33) thresholds were set as exploratory-stage parameters (alpha setting only, no corrections for multiple testing were applied) in the comparison of differential abundance of miRNAs in different groups of samples. All data analysis was performed on a subset of samples (*N* = 10, six HPV-positive and four HPV-negative tonsillar tumors) with high tumor fraction (≥60 % of tumor cells). Only the miRNAs differentially expressed in at least 3/5 (60 %) samples with a valid (measured) result (except for groups with two samples where all samples had to have a measured result) were considered for further analyses.

In order to determine new variables reflecting information on the differentially expressed miRNAs in relation to patient prognosis, tumor size, patient age, and grading, we applied factor analysis allowing for the reduction of the number of variables, taking advantage of a relatively large set of miRNAs analyzed across ten patients with tonsillar tumor only. The set of data was reduced and the miRNAs not expressed in two or more of ten analyzed samples were excluded. Altogether, 368 miRNAs, prognosis, size of tumor, age, and grading were evaluated. Additional available characteristics, such as tumor stage, nodal status, smoking status, and alcohol consumption status, were identical in all or almost all subjects. This analysis was done using IBM SPSS v23.

### Technical validation of microarray data

Selected miRNAs (miR-21, miR-205, let-7b, miR-24, miR-126, miR-378, miR-141, miR-200c, miR-146b, miR-191, and miR-484) which were found differentially expressed by the microarrays in tonsillar samples were further validated by the individual RT qPCR using the TaqMan® MicroRNA Assays (Life Technologies, USA) on the same set of samples. First, 10 ng of total RNA was reverse transcribed using the TaqMan® MicroRNA Reverse Transcription Kit (Life Technologies, USA) and predesigned primers for each individual TaqMan® MicroRNA Assay according the manufacturer's instructions. RNU48 was used as the endogenous control. For each sample, qPCR reaction was done in triplicate and consisted of 2 μl cDNA, 1× TaqMan® MicroRNA Assay, 5 μl of TaqMan® Universal PCR Master Mix, no AmpErase UNG (Life Technologies, USA), and nuclease-free water. The cycling conditions were 2 min at 50 °C and 10 min at 95 °C, followed by 40 cycles of denaturation at 95 °C for 15 s and annealing at 60 °C for 1 min. The pool of unaffected tonsils was used as a calibrator. The 2^-ΔΔCt^ method was used for calculations of the fold change. The overall agreement between the microarray data and individual assay data was excellent with the exception of miR-378 where the directions of regulation were opposite.

### Confirmation of microarray results

The confirmation of the differentially expressed miRNAs was performed on a large set of 64 macrodissected FFPE tumor samples (46 HPV-positive tumors and 18 HPV-negative tumors). The miRNA expression was assayed by individual RT qPCR using the TaqMan® MicroRNA Assays (Life Technologies, USA) as described above. As a calibrator, the pool of total RNA from unaffected tonsils were used. RNU48 was used as the endogenous control. The 2^-ΔΔCt^ method was used for calculations of the fold change. The cut-off fold change was set as for the arrays to +/− 1.33.

## Results

### Characterization of patients and samples

Demographic and clinical pathological parameters of patients with tonsillar tumors are summarized in Additional file 1: Table S1. The mean age of patients with tonsillar squamous cell carcinomas (TSCC) was 54.8 years and most of them were males (91.3 %). All HPV-positive tonsillar tumors contained HPV type 16 and expressed HPV16 E6 mRNA. The analysis of HPV genome status in HPV-positive tonsillar samples was done as published recently [25], with 43 % of the samples containing extrachromosomal HPV DNA, 14 % both extrachromosomal and integrated HPV DNA, and 36 % integrated HPV DNA. One sample yielded inconclusive results. All cervical tumors were HPV16-positive, whereas the non-malignant cervical tissues were HPV-negative. The majority of cervical tumors contained integrated HPV DNA.

### Global miRNA profiling in the model system

**Fig. 1:**
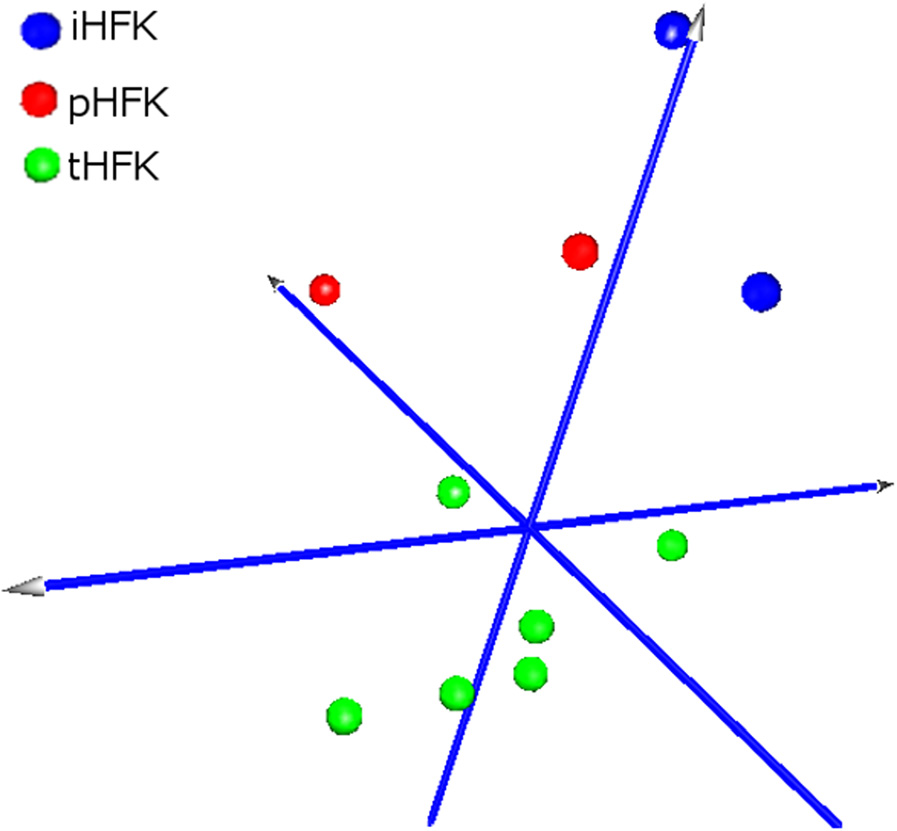
Principal component analysis plot. Visualization of miRNA expression in model systems. Primary human keratinocytes (pHFK); human keratinocytes immortalized by human telomerase gene (iHFK); HPV immortalized keratinocytes (tHFK)

MiRNA expression profiling was done in the model system of primary and immortalized human keratinocyte clones. As illustrated in Fig. 1, each group of the analyzed clones exhibited a very specific miRNA expression profile in principal component analysis (PCA).

The miRNA expression profiles of human keratinocyte clones were evaluated as a model system for clinical samples. As an analogy to the comparison of HPV-positive and/or HPV-negative tonsillar tumors with normal tissues, HPV16 immortalized keratinocyte clones and/or keratinocyte clones immortalized by the human telomerase gene and primary keratinocytes were used. In keratinocyte clones immortalized by HPV16, 39 miRNAs were differentially expressed whilst in keratinocyte clones immortalized by human telomerase gene, 30 miRNAs were found. MicroRNAs identified as differentially expressed with the *P*-value (*P* < 0.05) and fold-change (FC> 1.33) in the corresponding comparisons are listed in Additional files 2 and 3: Tables S2 and S3. MiRNAs miR-135b-3p, miR-146b-5p, miR-205-5p, miR-425-3p, miR-625-3p, and miR-485-3p which are involved in signaling and cell migration connected with epithelial to mesenchymal transition, tumor invasion, and metastasis, were detected in both the comparison of HPV immortalized keratinocytes with primary keratinocytes and the comparison of telomerase immortalized clones with primary keratinocytes.

### Global miRNA profiling in tumors and controls

In human tissues, non-censored PCA showed clear separation of the two groups of samples: normal HPV-negative cervical samples and non-malignant tonsillar tissue samples, suggesting the tissue specificity of the miRNA expression profile (Additional file 4: Figure S1). Clear group separation based on the miRNA expression profiles was also detected for the group of tumor samples and samples of normal tissue from the same anatomical location (Additional file 5: Figures S2A and B).

**Fig. 2:**
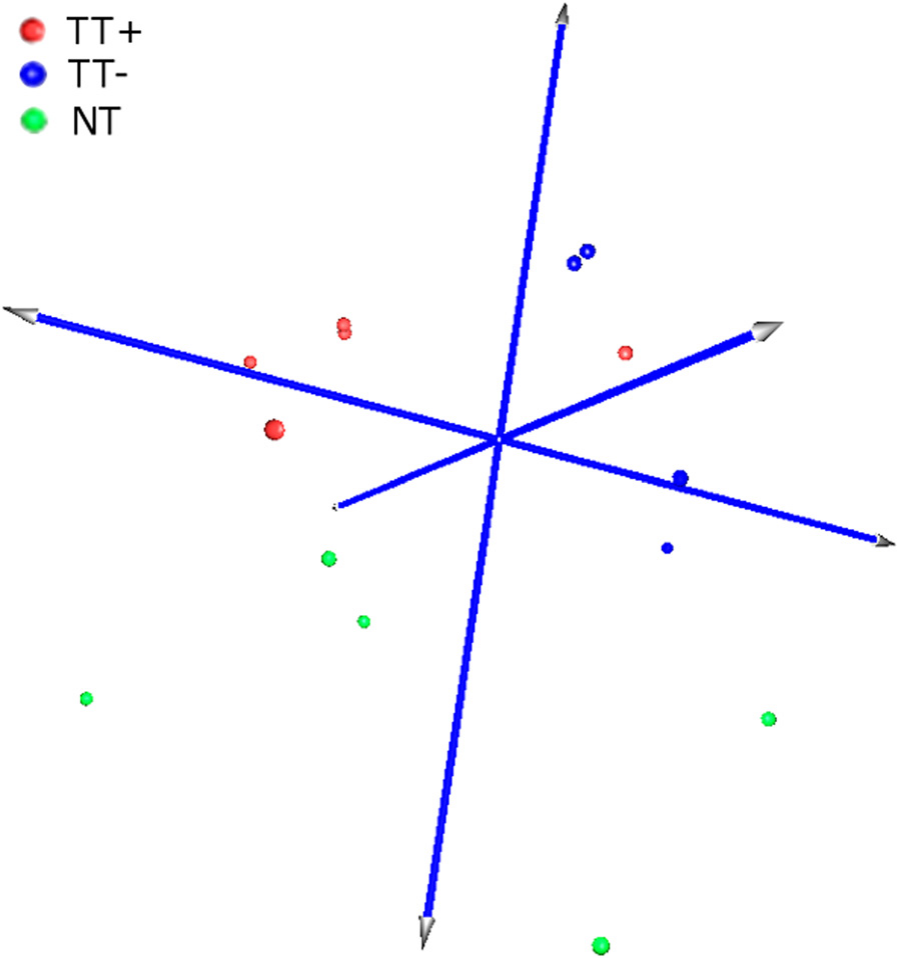
Principal component analysis plot. Visualization of miRNA expression of tonsillar tumors and non-malignant tissues. HPV-positive tonsillar tumors (TT+); HPV-negative tonsillar tumors (TT-); non-malignant tonsillar tissue (NT)

Figure 2 summarizes the miRNA expression of HPV-positive tonsillar tumors, HPV-negative tonsillar tumors, and non-malignant tonsillar tissues. We included only FF samples with more than 60 % of tumor cells which show much better separation in comparison to all samples. We found that tumor sample homogeneity (rather than sample size) is important for the robustness of miRNA expression analysis as separation is less clear if all samples irrespective of tumor cell content are visualized (data not shown).

The comparison of the miRNA expression profiles of tonsillar tumors and normal tonsillar tissues revealed 46 upregulated and 31 downregulated miRNAs in HPV-positive tumors (listed in Additional file 6: Table S4) and 42 upregulated and 42 downregulated miRNAs in HPV-negative tumors (listed in Additional file 7: Table S5).

**Fig. 3:**
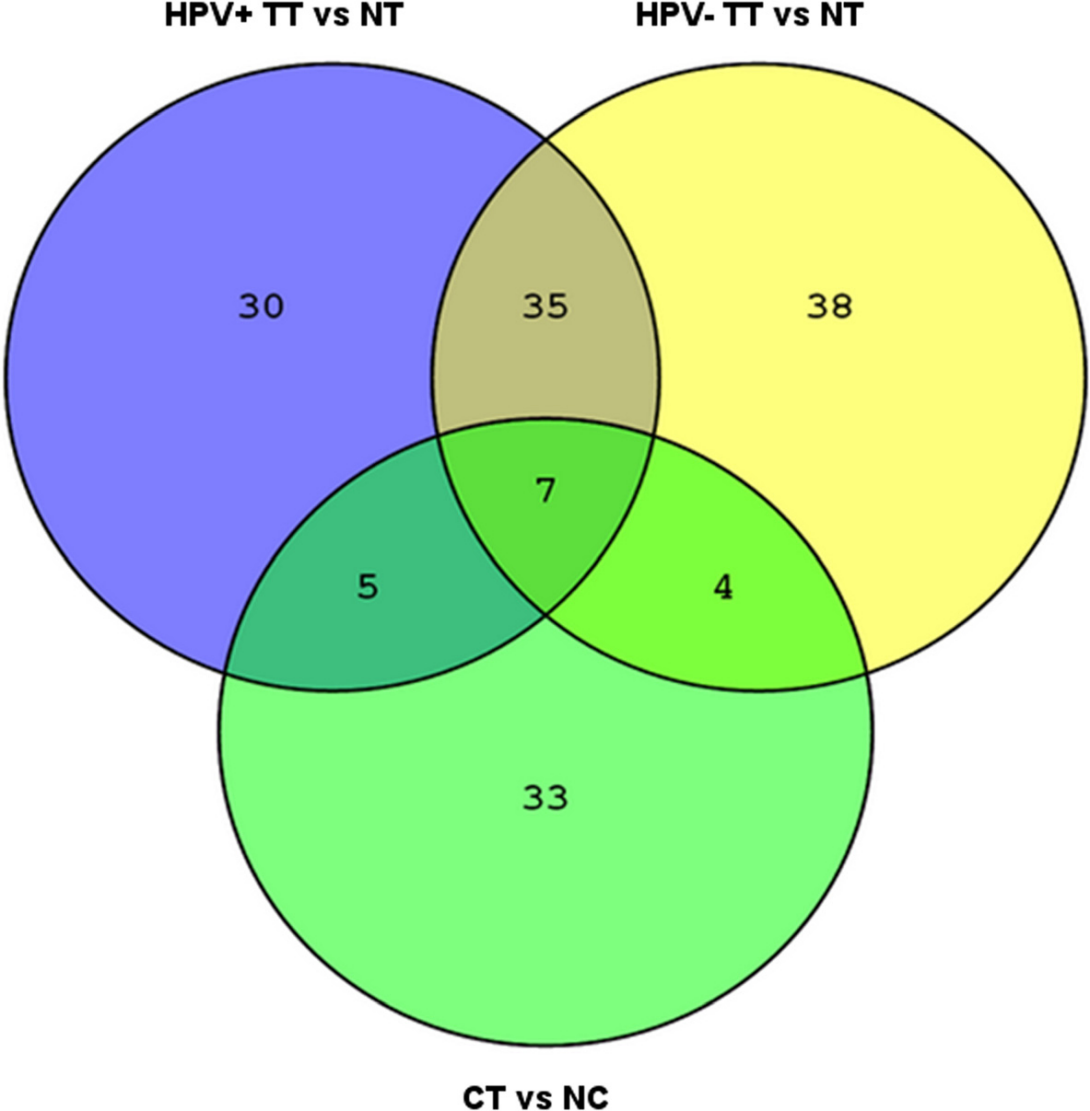
Venn diagram. Visualization of the number of group-specific miRNAs. HPV-positive tonsillar tumors (TT+); normal tonsils (NT); HPV-negative tonsillar tumors (TT-); cervical tumors (CT); normal cervix (NC)

To illustrate and identify the miRNAs specific for HPV-induced tumors and tonsillar tumors associated with or independent of HPV, a Venn diagram was constructed (Fig. 3). Altogether, we identified five miRNAs specific for HPV-induced malignancies, the so-called HPV-core miRNAs, common for HPV-positive TSCC and cervical tumors (listed in Table 2). One of these five miRNAs was inversely expressed in tonsillar and cervical tumors. The group of miRNAs specific for HPV-positive tonsillar tumors encompassed 30 miRNAs, and 38 miRNAs were exclusively specific for HPV-negative tonsillar tumors (all miRNAs listed in Table 3). Additionally, 35 miRNAs were common to both HPV-positive and HPV-negative tonsillar tumors which seem to be characteristic for the anatomical location of head and neck cancer (listed in Additional file 8: Table S6).

**Table 2:** List of HPV core miRNAs

Probe name	miRNA family	TT+ versus NT^a^		CT versus NC^b^	
		Fold change	*p*-value	Fold change	*p*-value
miR-141-3p	mir-8	6.175	0.000197	17.039	0.002
miR-15b-5p	mir-15	3.072	0.034	10.659	0.045
miR-200a-3p	mir-8	8.173	0.000269	6.481	0.005
miR-302c-3p	mir-302	29.111	0.093	−1862.248	0.005
miR-9-5p	mir-9	24.460	0.001	177.296	0.018

^a^ *TT+* HPV-positive tonsillar tumors, *NT* normal tonsils

^b^ *CT* cervical tumors, *NC* normal cervix

**Table 3:** List of group-specific miRNAs derived from the Venn diagram

Exclusively TT+ specific^a^			Exclusively TT- specific^a^		
Probe name	miRNA family	FC^b^	Probe name	miRNA family	FC^b^
hsa-miR-125b-2-3p	mir-10	21.689	hsa-miR-431-5p	mir-431	37.879
hsa-miR-147b	mir-147	16.057	hsa-miR-517c-3p	mir-515	12.679
hsa-miR-523-3p	mir-515	10.983	hsa-miR-485-3p	mir-485	7.305
hsa-miR-542-3p	mir-542	9.273	hsa-miR-623	mir-623	6.535
hsa-miR-10a-5p	mir-10	4.513	hsa-miR-34c-5p	mir-34	4.851
hsa-miR-202-3p	mir-202	3.694	hsa-miR-221-3p	mir-221	4.505
hsa-miR-34a-3p	mir-34	2.854	hsa-miR-517a-3p	mir-515	4.327
hsa-miR-34a-5p	mir-34	2.574	hsa-miR-193b-3p	mir-193	2.966
hsa-miR-21-5p	mir-21	2.494	hsa-miR-193a-5p	mir-193	2.620
hsa-miR-335-5p	mir-335	2.378	hsa-miR-223-3p	mir-223	2.518
hsa-miR-18b-5p	mir-17	2.338	hsa-miR-323a-3p	mir-154	2.391
hsa-miR-130b-3p	mir-130	2.180	hsa-miR-1260a	mir-1260a	2.202
hsa-miR-197-3p	mir-197	2.077	hsa-miR-106b-3p	mir-17	2.072
hsa-miR-579-3p	mir-548	1.945	hsa-miR-151a-3p	mir-28	1.735
hsa-miR-132-3p	mir-132	1.699	hsa-let-7d-5p	let-7	−1.389
hsa-miR-214-5p	mir-214	−2.571	hsa-miR-30b-5p	mir-30	−1.461
hsa-miR-30e-3p	mir-30	−2.934	hsa-miR-487a-3p	mir-154	−1.474
hsa-miR-425-3p	mir-425	−3.147	hsa-let-7e-5p	let-7	−1.554
hsa-miR-500a-3p	mir-500	−3.333	hsa-let-7b-5p	let-7	−1.582
hsa-miR-126-5p	mir-95	−4.049	hsa-miR-564	mir-564	−1.590
hsa-miR-145-3p	mir-145	−4.805	hsa-miR-629-3p	mir-629	−1.616
hsa-miR-1247-5p	mir-1247	−5.233	hsa-miR-125b-5p	mir-10	−1.920
hsa-miR-505-5p	mir-505	−5.344	hsa-miR-100-5p	mir-10	−2.106
hsa-miR-30a-3p	mir-30	−5.981	hsa-miR-30d-5p	mir-30	−2.145
hsa-miR-136-3p	mir-136	−6.714	hsa-miR-26b-3p	mir-26	−2.369
hsa-miR-486-5p	mir-486	−7.824	hsa-miR-29a-3p	mir-29	−2.397
hsa-miR-1179	mir-1179	−8.498	hsa-miR-130b-5p	mir-130	−2.647
hsa-miR-1	mir-1	−10.729	hsa-miR-642a-5p	mir-642	−2.768
hsa-miR-575	mir-575	−11.791	hsa-miR-328-3p	mir-328	−2.806
hsa-miR-133a-3p	mir-133	−24.685	hsa-miR-744-3p	mir-744	−2.998
			hsa-miR-520d-3p	mir-515	−3.135
			hsa-miR-99a-5p	mir-10	−3.404
			hsa-miR-29c-3p	mir-29	−3.474
			hsa-miR-139-3p	mir-139	−3.666
			hsa-miR-885-5p	mir-885	−3.725
			hsa-miR-138-5p	mir-138	−12.185
			hsa-miR-142-3p	mir-142	−15.980
			hsa-miR-150-5p	mir-150	−17.261

^a^ *TT+* HPV-positive tonsillar tumors, *TT-* HPV-negative tonsillar tumors

^b^ *FC* fold change

### Comparison of miRNA profiles in clinical samples and the model system

The aim of our study was also the comparison of miRNA expression in clinical samples and in the model system of keratinocyte clones. HPV-positive tonsillar tumors and HPV-immortalized keratinocyte clones overlapped in three miRNAs (Table 4) specific to either group. MiR-335-5p and miR-579-3p were upregulated in both groups, whereas miR-126-5p was upregulated in clinical samples and downregulated in the model system. We also identified seven additional miRNAs common to these two groups, but they were significantly deregulated either in HPV-negative tonsillar tumors in comparison with normal tonsils or in keratinocyte clones immortalized by human telomerase (hTERT) in comparison with primary keratinocytes, so they seemed not to be directly influenced by the presence of HPV. HPV-negative tumors and hTERT-immortalized keratinocyte clones overlap in three miRNAs specific to either group and nine additional miRNAs which were common also to other groups. We detected miRNA-328-3p which was upregulated in both groups. Inverse patterns of expression regulation of miR-34c-3p and miR-885-5p were found in the model system and clinical samples.

**Table 4:** List of significantly deregulated (*P* < 0.05) miRNAs in clinical samples vs. the model system

Probe name	miRNA family	tHFK vs. pHFK^a^	TT+ vs. NT^b^
		Fold change	Fold change
hsa-miR-335-5p	mir-335	8.361	2.378
hsa-miR-579-3p	mir-548	7.921	1.945
hsa-miR-126-5p	mir-95	2.580	−4.049
Probe name	miRNA family	iHFK vs. pHFK^a^	TT- vs. NT^b^
		Fold change	Fold change
hsa-miR-328-3p	mir-328	−22.020	−2.806
hsa-miR-34c-5p	mir-34	−16.419	4.851
hsa-miR-885-5p	mir-885	48.377	−3.725

^a^ *tHFK* HPV-immortalized human foreskin keratinocyte clones, *pHFK* primary human foreskin keratinocyte clones, *iHFK* hTERT-immortalized human keratinocyte clones

^b^ *TT+* HPV-positive tonsillar tumors, *TT-* HPV-negative tonsillar tumors

In the groups of HPV-positive tonsillar tumors and HPV-immortalized keratinocyte clones, we also evaluated the influence of HPV status on the changes in the miRNA expression profile. HPV-positive tumor tissues which contain extrachromosomal, integrated, or mixed HPV DNA differ in the miRNAs expression profiles. A similar situation was observed in the model system of keratinocyte clones. However, we found no overlap in the list of miRNAs differentially expressed in HPV-positive tumors and in HPV-immortalized keratinocytes with a particular form of HPV DNA, but the differentially expressed miRNAs were found to target the same genetic pathways.

### Confirmation of microarray results

To confirm the results of differentially expressed miRNAs revealed by miRNA arrays, we selected nine specific miRNAs and confirmed their expression in a set of 64 FFPE samples by individual TaqMan miRNA assays. The miRNAs were selected based on the fold change and relevance in the literature. From the group of HPV core miRNAs, we selected miR-9, miR-141, and miR-200a which were upregulated in both groups of HPV-associated tumors (Additional file 9: Figure S3). From the group of the miRNAs specific for HPV-positive tonsillar tumors, the upregulated miR-125b-2*, miR-21, and miR-335 were selected (Additional file 10: Figure S4). MiR-335 was also upregulated in the model system of HPV-immortalized keratinocyte clones. Next, miR-221 upregulated in a group of HPV-negative tonsillar tumors was analyzed (Additional file 11: Figure S5). Finally, miR-20b exemplified the miRNAs upregulated in HPV-positive tonsillar tumors and downregulated in HPV-negative tonsillar tumors, and miR-210 exemplified those upregulated in tonsillar tumors of either etiology (Additional file 12: Figure S6).

**Fig. 4:**
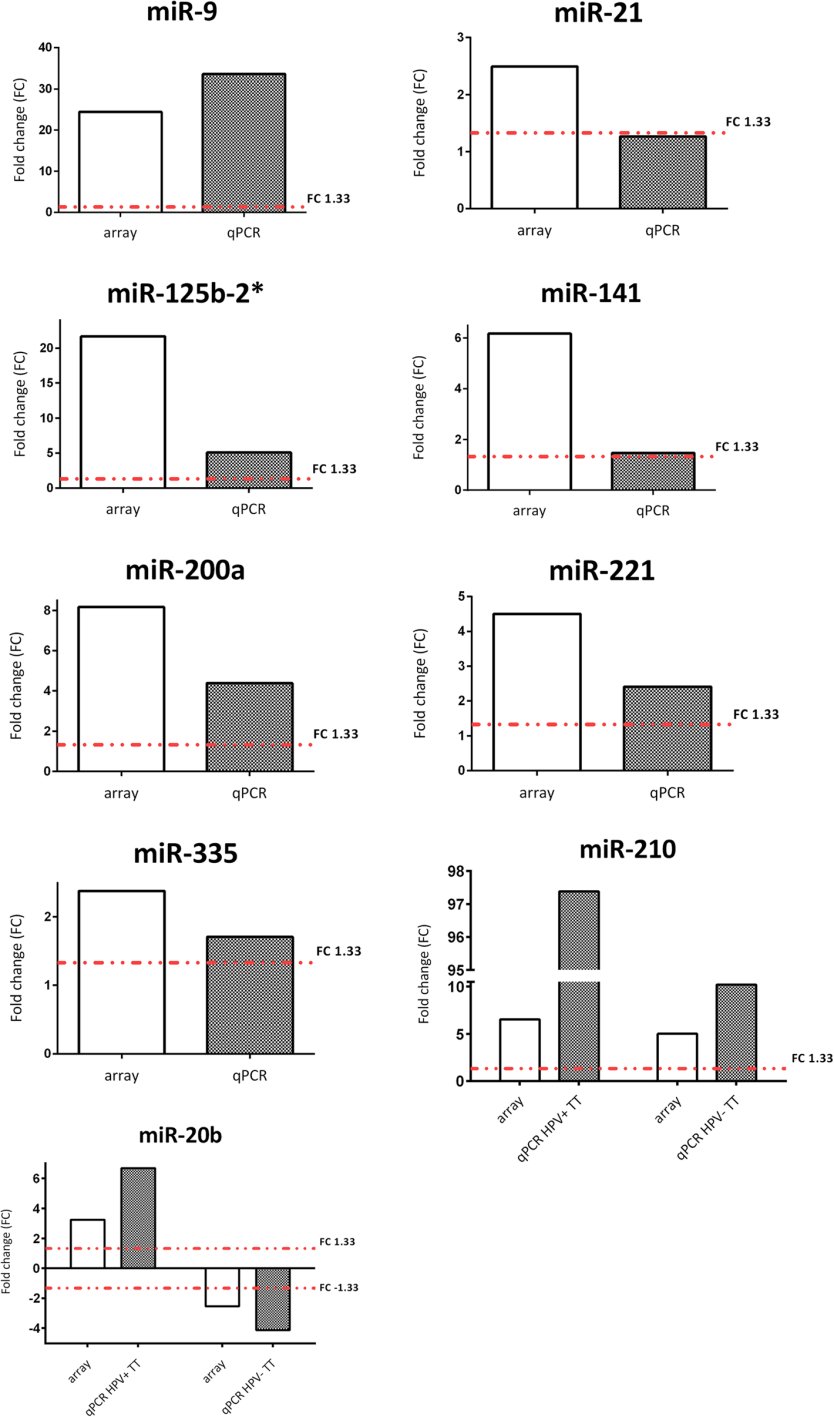
Comparison of fold change for particular miRNAs between arrays and qPCR. HPV-positive tonsillar tumors (HPV+ TT), HPV-negative tonsillar tumors (HPV- TT). The fold change (FC) threshold was set to 1.33

The expression of all above-mentioned miRNAs was confirmed in a set of FFPE macrodissected tumors. The values of fold changes based on individual assays were then compared with the values obtained with miRNA arrays (Fig. 4). The cut-off fold change for significantly deregulated miRNAs was set as for arrays to +/− 1.33. All individually analyzed miRNAs showed concordance of the expression with the results obtained with arrays, with the exception of miR-21. Although the trend in expression was maintained, the upregulated expression of miR-21 was confirmed only in 50 % of samples, and the median of absolute fold change in all samples was equal to 1.27. The deregulated expression of the other miRNAs was confirmed (miR-9 in 100 %, miR-141 in 59 %, miR-200a in 87 %, miR-125b-2* in 89 %, miR-335 in 54 %, miR-221 in 67 %, miR-20b in 93 % of HPV-positive tumors and in 87 % of HPV-negative tumors, and miR-210 in 100 % of tumors of both etiologies).

### Prognostic miRNAs

The expression of 738 miRNAs was evaluated in ten tonsillar tumor samples. In order to evaluate if the analyzed miRNAs correspond with the clinical characteristics or patient prognosis, we performed the factor analysis. At first we compute the principal components and for them we made varimax rotation to obtain best view to data. The loading of obtained factor is displayed in Additional file 13: Figure S7, the components of the particular rotated factor are listed in Additional file 14: Table S7. For the analysis we used IBM SPSS v.23. One hundred percent of the variance can be explained by eight groups of factors and prognosis was the most strongly associated with miRNAs from group 5, which in total explains about 10.3 % of the variability. Twelve miRNAs in group 5 (miR-196b, miR-485-3p, miR-589, miR-324-3p, miR-342-3p, miR-92a1#, miR-155, miR-146b, miR-142-3p, miR-1260, miR-143, and miR-142-5p) have strong loading (absolute value ≥ 0.8).

## Discussion

While the incidence of head and neck tumors of non-viral etiology is declining, the head and neck tumors associated with human papillomavirus infection are steadily increasing [5, 30]. The presence of HPV remains the strongest prognostic factor in HNC compared to HPV-negative tumors [9, 31]. Thus, the task of advancing knowledge on molecular pathogenesis of the disease and identifying diagnostic and prognostic biomarkers remains of key importance. Our aim was to characterize the expression profiles of miRNAs in well-characterized tonsillar tumors and define the miRNAs characteristic of HPV-associated or HPV-negative tonsillar tumors. Next, we wanted to compare the results from clinical samples with those obtained from the model system of isogenic primary human keratinocyte clones immortalized by HPV or human telomerase gene.

MicroRNA profiling in HNC has been reported in several studies, but the published data lack comparability, likely due to the anatomical heterogeneity of the studied tumors and differences in the methodological approaches. Tran et al. profiled miRNAs in a set of head and neck cancer cell lines [17], similarly to Chang et al. who have compared results from cell lines with the miRNA profiles from a small group of four tumor samples [32]. Afterwards, studies performed on tumor tissues from various locations have been published [18-20, 33]. However, none of these studies addressed a comparison between HPV-associated and HPV-independent tumors, although other studies analyzed the miRNA profiles in cell lines or clinical samples with regard to the HPV status. Wald et al. have studied the miRNA profiles in head and neck squamous cell carcinoma (HNSCC) cell lines [21], identifying a number of deregulated miRNAs and describing the influence of HPV oncogene E6 on the miRNA profiles. Lajer et al. analyzed a set of 51 oral and pharyngeal tumors in comparison to the normal tissues, reporting deregulation of 21 miRNAs in nine HPV-positive samples [22]. This study became extended to include a set of tonsillar and cervical tumors, and to identify miRNAs with roles in HPV-associated pathogenesis [23]. However, both these studies lacked confirmation of the transcriptionally active HPV infection. Hui et al. have identified several miRNAs associated with HPV status in a set of oropharyngeal carcinomas and suggested candidate miRNAs correlating with the patients’ clinical outcome [34]. Recently, Miller et al. have identified a miRNA subset in oropharyngeal carcinomas, validating the analysis in the clinical data from The Cancer Genome Atlas [35].

Here we analyzed the miRNA expression profiles of well-characterized tonsillar tumors and in a set of keratinocyte clones, providing a first-of-its-kind comparison of the miRNA profiles in clinical samples with regard to the presence of HPV, with a model system of keratinocyte clones. Keratinocyte clones immortalized by HPV16 and primary keratinocytes were used as the model system for the HPV-positive tumors and/or normal tissues, whilst the keratinocyte clones immortalized by human telomerase gene mimicked HPV-independent tumors.

When comparing the two types of immortalized keratinocyte clones against primary cells, in order to identify the miRNAs characteristic for immortalized cells regardless of the mode of immortalization, miR-135b-3p and miR-146b-5p were downregulated while miR-205-5p was upregulated in both types of immortalized keratinocyte clones. This is in agreement with other miRNA profiling studies of different carcinomas and cell lines including HNC, and these miRNAs have been shown to participate in the signaling pathways and cell migration pathways connected with epithelial to mesenchymal cell transition (EMT), tumor invasion, and metastasis [36–38]. Next, we identified the miRNAs specific either for the HPV-immortalized keratinocyte clones or the hTERT-immortalized clones. The most upregulated miRNA in HPV-immortalized clones was miR-454-5p, reported previously as upregulated in human colorectal cancer cells [39]. Another highly upregulated miRNA was the tumor suppressor-like miR-335, linked to longer survival in cervical cancer patients [40, 41]. The most downregulated miRNAs in HPV-immortalized keratinocyte clones were miR-33a-5p and miR-133b. Wong et al. have demonstrated the tumor suppressor role of miR-133b in oral cell lines through the dysregulation of pyruvate kinase type M2 (PKM2) [42]. MiRNA-146a, here observed to be downregulated in HPV-immortalized keratinocytes, is also regulated by HPV oncoproteins, indirectly through the activation of c-*Myc* oncogene via viral E7 protein (reviewed by [43]).

The most upregulated miRNAs in hTERT-immortalized keratinocyte clones were less-studied miR-627-5p and miR-885-5p, although miR-885-5p has been reported to act as the post-transcriptional regulator of *CASP3* expression which has anti-apoptotic and carcinogenic effects [44]. MiR-199a-3p and miR-146b-5p were the most downregulated miRNAs in hTERT-immortalized clones. Several studies focused on the tumor type-dependent function of miR-199-3p in tumors and cell lines have found that its downregulation enhances proliferation, invasiveness, and adhesion [45] and it is a predictor of worse prognosis in patients with osteosarcoma [46].

In this study, we showed that each group of tumors has a specific miRNA profile. The most upregulated miRNAs in HPV-positive tonsillar tumors were miR-125b-2-3p and miR-147b while the most downregulated were miR-133a-3p and miR-575. MiR-125b-1 and miR-125b-2 originated from independent precursors located in different chromosomal loci, but their targets are identical. In contrast to our data, Nakanishi et al. have revealed the loss of miR-125b-1 to contribute to head and neck cancer development [47] and Henson et al. have reported decreased expression of miR-125b in oral cancer cells [48]. In the context of HPV infection, Nuovo et al. have observed that miR-125b has a role in productive HPV infection and that its upregulation leads to the reduction in viral DNA [49]. However, other miRNAs found to be upregulated in HPV-positive tumors in our study were also identified to play a role in cancer (e.g. miR-34a, miR-21, miR-10a, or some mir-30 family members). MiR-133a, tumor suppressor miRNA downregulated in several types of cancer including HNSCC, was also downregulated in our set of HPV-positive tumors [50–52]. MiR-133a has been shown to be involved in inhibition of cell proliferation, migration and invasion in HNSCC cell lines [53, 54].

In HPV-negative tumors, miR-431-5p and miR-517c-3p were the most upregulated and miR-150-5p and miR-142-3p the most downregulated miRNAs. Dysregulation of miR-150 has been demonstrated in a number of solid tumors (reviewed in [55]). Additionally, miR-485, miR-34c, miR-221, or miR-193a and miRNAs from the mir-10 or let-7 families identified as deregulated in HPV-negative tumors in our study participate in the regulation of proliferation, apoptosis, and invasion, and have been proposed as a prognostic indicators in patients with solid cancers [56–60].

As mentioned above, only three studies analyzed the miRNA profiles in head and neck tumors with regard to the HPV status. Lajer et al. addressed the miRNA profiles in HNC and compared the miRNA profiles of HPV-positive and HPV-negative HNSCC and cervical carcinomas, identifying a group of HPV-associated core miRNAs [22, 23]. Negligible overlap with their results was found in our study most likely due to the heterogeneity in the analyzed samples in Lajer´s study. Lajer et al. compared cervical tumors with a pool of HPV-positive tonsillar and pharyngeal carcinomas while in our study only well-characterized tonsillar tumors were evaluated. Our results do agree with theirs only in the identification of miR-21, the most commonly elevated miRNA in cancers. In agreement with the study of Hui et al. [34], we identified miR-9 as associated with the HPV status since it was upregulated in both tonsillar and cervical tumors. Recent studies have shown miR-9 to be involved in the pathways regulating metastasis [61]. Liu et al. [62] have shown that HPV-induced activation of miR-9 leads to the increase of cell motility through downregulation of genes involved in the pathways of cell migration. MiR-9 has also been considered as HPV-associated by Miller et al. using the bioinformatics analysis [35].

Despite the small overlap in the identified HPV core miRNAs with Lajer´s study the other HPV core miRNAs identified in our study have been previously mentioned as players in cancer development. Myklebust et al. have reported miR-15b to be strongly associated with the expression of several E2F-related genes [63] and its expression to be reduced after HPV16 E7 knockdown. MiR-141 has been found to be overexpressed in HPV-positive cervical carcinomas [64]. Members of the mir-8 family, which includes also miR-200a, are the major regulators of the EMT pathway, primarily targeting transcriptional factors ZEB1 and ZEB2 [65]. MiR-302c-3p inversely expressed in tonsillar and cervical tumors in our study has been shown before as miRNA inhibiting the tumor growth [66].

The aim of our study was to compare the miRNA expression profiles in clinical samples and in the model system of keratinocyte clones. The overlap between particular clinical samples and the model system was very small. The miRNA profiles of HPV-positive tonsillar tumors and HPV-immortalized clones overlap in three miRNAs while one was inversely expressed in clinical samples and in keratinocyte clones. HPV-negative tumors and hTERT-immortalized clones overlap also in three miRNAs. Two were expressed inversely in the two groups, miR-328-3p was downregulated in both groups, and decreased expression of this miRNA has also been reported in other carcinomas [67, 68].

Because we have done the detailed analysis of the HPV genome status in our HPV-positive tonsillar tissues and keratinocyte clones, we were able to evaluate the differences in the expression miRNA profiles in relation to the status of the viral genome. Tumor tissues which contain either extrachromosomal, integrated, or mixed form of HPV genome differ in their miRNA expression profiles, as was documented in the model system as well. Even though there is no overlap in the lists of miRNAs in tumors and HPV-immortalized keratinocytes regarding the genome status, the differentially expressed miRNAs were found to target the same pathways; pathways in cancer, p53 signaling, cell cycle regulation, and PI3K-Akt signaling pathways.

We confirmed the expression of nine specific miRNAs by individual TaqMan assays in a set of 64 FFPE samples. For some of these miRNAs the deregulated expression was not confirmed in all samples; miR-21 was deregulated in 50 % of FFPE samples, miR-335 in 54 %, miR-141 in 59 % and miR-221 in 67 %. This is most likely due to the difference in the number of tumor cells in the samples. For arrays FF samples with >60 % of tumor cells were used while for RT qPCR macrodissected FFPE samples containing only the tumor cells were analyzed. The expression profiles of less deregulated miRNAs can be easily influenced by the presence of non-malignant cells. The deregulation of all others miRNAs was confirmed in more than 80 % of FFPE samples.

Patients with tonsillar tumors included in our study were followed up for a long time, and we wanted to explore the relationship between the miRNA expression and patient prognosis. Since the number of variables was high and the number of samples with the completed analysis of 738 miRNAs was low, factor analysis was performed. Twelve miRNAs in this group (miR-196b, miR-485-3p, miR-589, miR-324-3p, miR-342-3p, miR-92a-1#, miR-155, miR-146b, miR-142-3p, miR-1260, miR-143, and miR-142-5p) seem to be influential on patient prognosis. The majority of these miRNAs have been reported as prognostic markers in different types of cancers, while miR-324-3p, miR-155, miR-142-3p, and miR-143 have been identified as prognostic indicators in head and neck tumors of different locations [69–73]. The expression levels of these miRNAs merit further validation in a larger set of tonsillar tumors.

## Conclusions

In conclusion, we characterized the miRNA expression profiles of HPV-associated and HPV-independent tumors and compared the results with those obtained in the primary and immortalized human keratinocyte clones. Our data show that the miRNA profiles differ between tissues of different anatomical origin. Tumor tissues exhibited site-specific miRNA expression profiles, but the heterogeneity of the miRNA profiles in tonsillar tumors was much larger than in cervical tumors. We established that tumor sample homogeneity (rather than sample size) is important for the robustness of the miRNA expression analysis. We identified miRNAs specific for either tonsillar carcinomas or HPV-associated tumors and the results were compared with the data from the human keratinocyte clone model. The expression levels of the selected miRNAs were confirmed in a larger set of tonsillar tumor samples. Finally, we evaluated the differentially expressed miRNAs in relation to patient prognosis. Our results warrant further confirmation in a larger set of samples to allow future in-depth investigations of the role played by particular miRNAs in head and neck tumorigenesis.

## Additional files

Additional file 1: Table S1. Demographic characteristics and clinical-pathological parameters of patients with tonsillar tumors (and available fresh frozen specimens). (XLS 39 kb) Additional file 2: Table S2. Significant (*P* < 0.05) miRNAs differentially expressed in HPV16 immortalized keratinocytes (tHFK) in comparison to primary human keratinocytes (pHFK). (XLS 39 kb) Additional file 3: Table S3. Significant (*P* < 0.05) miRNAs differentially expressed in keratinocytes immortalized by human telomerase gene (iHFK) in comparison to primary human keratinocytes (pHFK). (XLS 38 kb) Additional file 4: Figure S1. Principal component analysis plot. Visualization of miRNA expression in normal HPV-negative cervical tissues (NC, blue) and in non-malignant HPV-negative tonsillar tissues (NT, green). (TIF 1057 kb) Additional file 5: Figure S2. Principal component analysis plot. Visualization of different miRNA expression in tumor and normal tissues of the same anatomical location. A - Tonsillar tumors (TT, red) versus non-malignant tissues (NT, green). B - Cervical tumors (CT, green) versus normal tissues (NC, blue). (TIF 1553 kb) Additional file 6: Table S4. Significant (*P* < 0.05) miRNAs differentially expressed in HPV-positive tonsillar tumors in comparison to non-malignant tonsillar tissues. (XLS 43 kb) Additional file 7: Table S5. Significant (*P* < 0.05) miRNAs differentially expressed in HPV-negative tonsillar tumors in comparison to non-malignant tonsillar tissues. (XLS 43 kb) Additional file 8: Table S6. Significantly (*P* < 0.05) expressed miRNAs common to both HPV-positive and HPV-negative tonsillar tumors. (XLSX 12 kb) Additional file 9: Figure S3. Expression values of HPV core miRNAs in miRNA arrays. Fold change >1.33, *P*-value <0.05. HPV-positive tonsillar tumors (HPV+ TT), normal tonsils (NT), cervical tumors (CT), normal cervical tissues (NC). (DOC 325 kb) Additional file 10: Figure S4. Expression values of miRNAs differentially expressed in HPV-positive tonsillar tumors on miRNA arrays. Fold change >1.33, *P*-value <0.05. HPV-positive tonsillar tumors (HPV+ TT), normal tonsils (NT), HPV-immortalized keratinocytes clones (tHFK), primary keratinocytes clones (pHFK). (DOC 166 kb) Additional file 11: Figure S5. Expression values of miR-221 differentially expressed in HPV-negative tonsillar tumors on miRNA arrays. Fold change >1.33, *P*-value <0.05. HPV-negative tonsillar tumors (HPV- TT), normal tonsils (NT). (DOC 123 kb) Additional file 12: Figure S6. Expression values of miR-210 and miR-20b differentially expressed in tonsillar tumors of both etiologies on miRNA arrays. Fold change >1.33, *P*-value <0.05. HPV-positive tonsillar tumors (HPV+ TT), HPV-negative tonsillar tumors (HPV- TT), normal tonsils (NT). (DOC 519 kb) Additional file 13: Figure S7. Screenplot showing the loading of the factors obtained by factor analysis. (TIF 920 kb) Additional file 14: Table S7. Rotated Component Matrix computed by Rotation Method: Varimax with Kaiser Normalization showing the components of a particular factor sorted by absolute value of loading for each variable. (XLS 146 kb)
