# Supplementary figures and images for: Comparison of the miRNA profiles in HPV-positive and HPV-negative tonsillar tumors and a model system of human keratinocyte clones
Source: BMC Cancer. 2016 Jul 4;16:382. doi: 10.1186/s12885-016-2430-y (PMC4932682; doi:10.1186/s12885-016-2430-y)

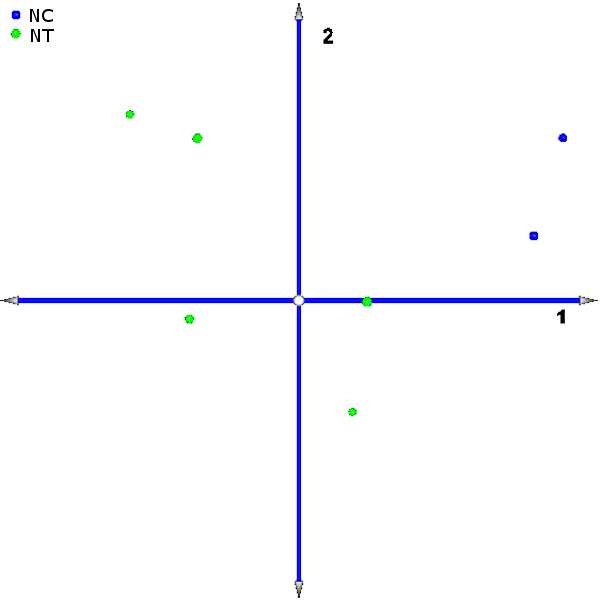

Supplement: Additional file 4: Figure S1. — Principal component analysis plot. Visualization of miRNA expression in normal HPV-negative cervical tissues (NC, blue) and in non-malignant HPV-negative tonsillar tissues (NT, green). (TIF 1057 kb) [file 12885_2016_2430_MOESM4_ESM.tif]

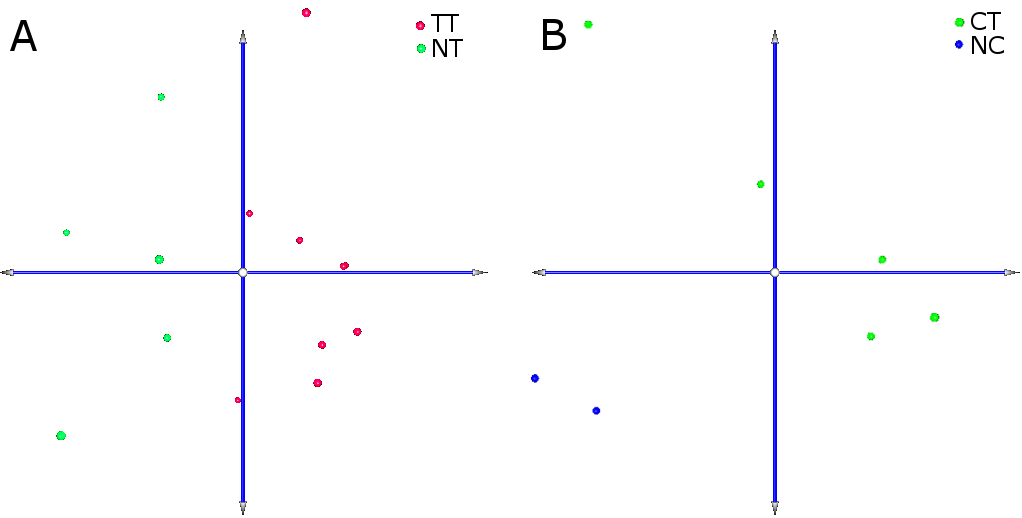

Supplement: Additional file 5: Figure S2. — Principal component analysis plot. Visualization of different miRNA expression in tumor and normal tissues of the same anatomical location. A - Tonsillar tumors (TT, red) versus non-malignant tissues (NT, green). B - Cervical tumors (CT, green) versus normal tissues (NC, blue). (TIF 1553 kb) [file 12885_2016_2430_MOESM5_ESM.tif]

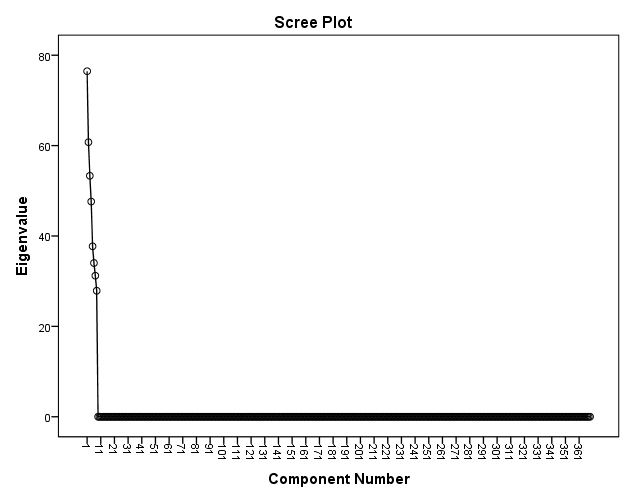

Supplement: Additional file 13: Figure S7. — Screenplot showing the loading of the factors obtained by factor analysis. (TIF 920 kb) [file 12885_2016_2430_MOESM13_ESM.tif]
