# Supplementary material for: Comparison of the miRNA profiles in HPV-positive and HPV-negative tonsillar tumors and a model system of human keratinocyte clones
Source: BMC Cancer. 2016 Jul 4;16:382. doi: 10.1186/s12885-016-2430-y (PMC4932682; doi:10.1186/s12885-016-2430-y)
Supplement: Supplementary file 5 [file 12885_2016_2430_MOESM10_ESM.doc]

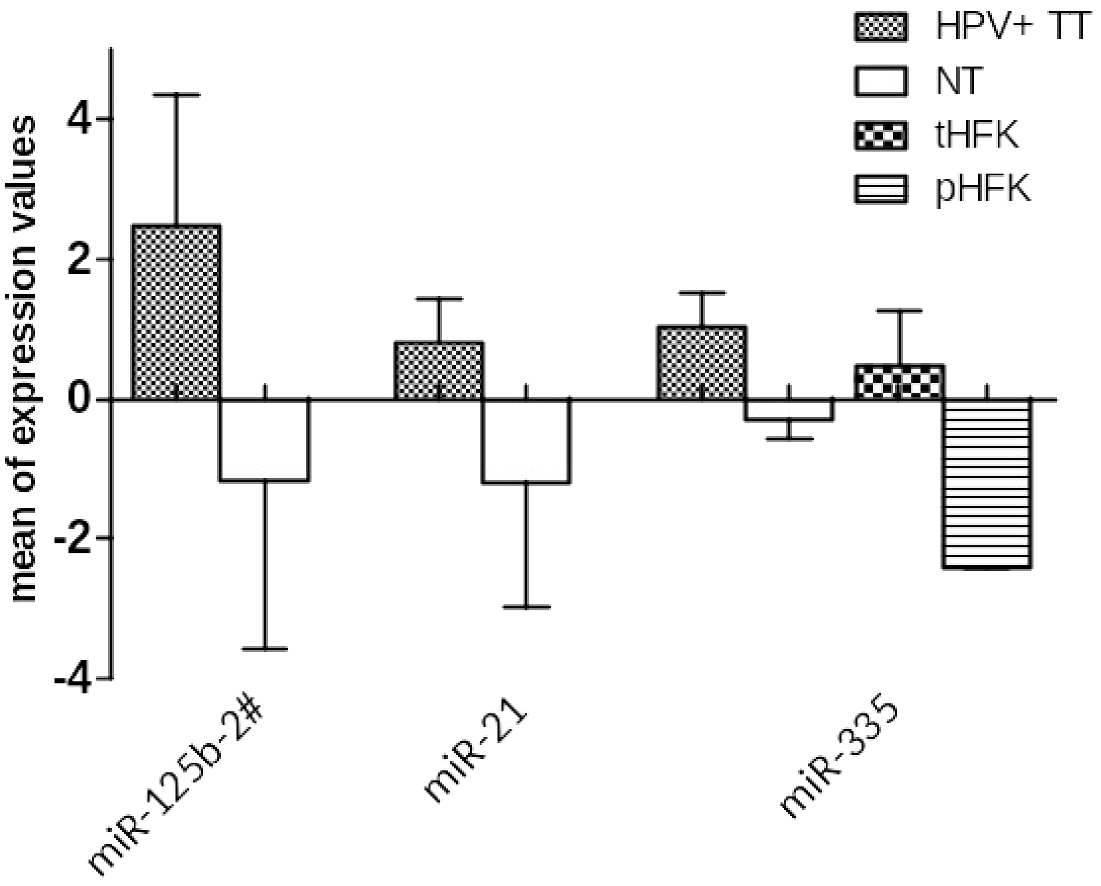
HPV+ TT
NT
tHFK
pHFK
mean of expression values
4
2
0
-2
-4
miR-125b-2#
miR-21
miR-335
