# Supplementary material for: Comparison of the miRNA profiles in HPV-positive and HPV-negative tonsillar tumors and a model system of human keratinocyte clones
Source: BMC Cancer. 2016 Jul 4;16:382. doi: 10.1186/s12885-016-2430-y (PMC4932682; doi:10.1186/s12885-016-2430-y)
Supplement: Supplementary file 8 [file 12885_2016_2430_MOESM9_ESM.doc]

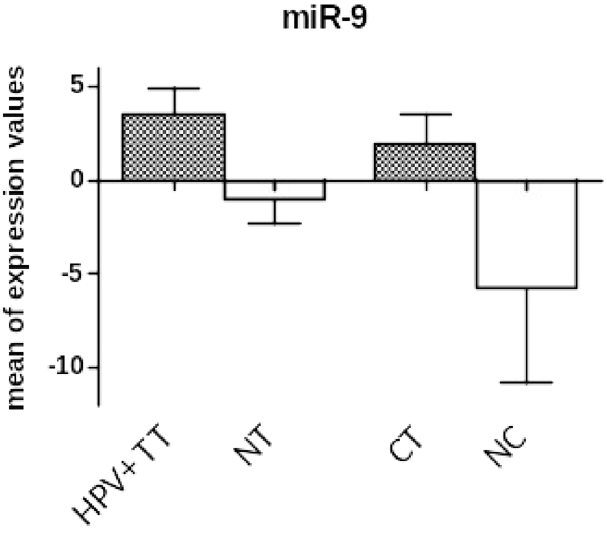
miR-9
mean of expression values
5
0
-5
-10
HPV+TT
NT
CT
NC

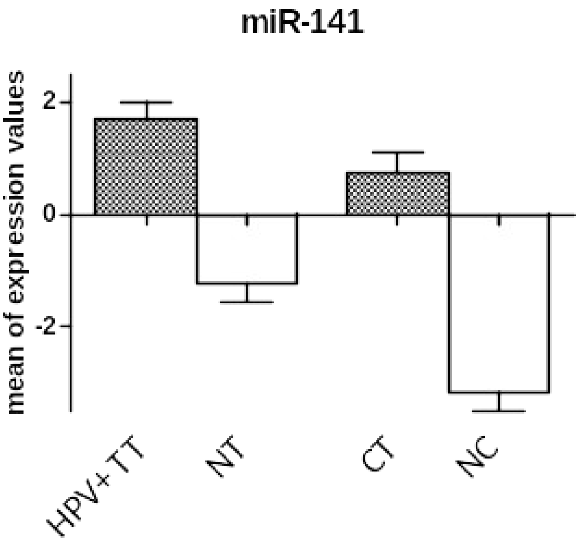
miR-141
mean of expression values
2
0
-2
HPV+TT
NT
CT
NC

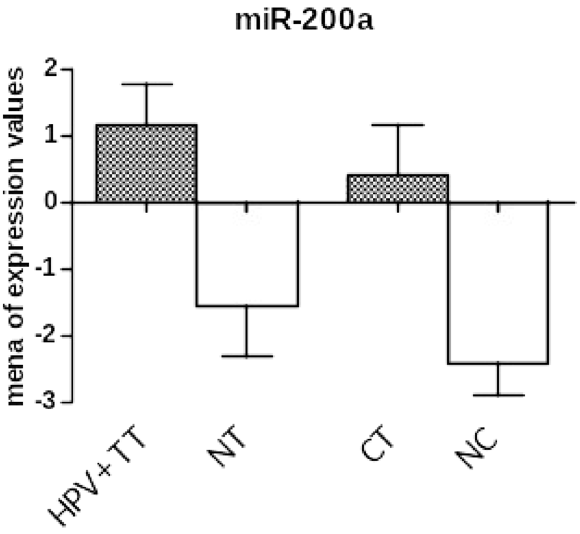
miR-200a
mena of expression values
2
1
0
-1
-2
-3
HPV+TT
NT
CT
NC
